# Antibacterial and Antibiotic-Potentiating Activities of Thirteen Cameroonian Edible Plants against Gram-Negative Resistant Phenotypes

**DOI:** 10.1155/2018/4020294

**Published:** 2018-09-10

**Authors:** Paul Nayim, Armelle T. Mbaveng, Brice E. N. Wamba, Aimé G. Fankam, Joachim K. Dzotam, Victor Kuete

**Affiliations:** Department of Biochemistry, Faculty of Science, University of Dschang, Cameroon

## Abstract

This work was designed to investigate the antibacterial activities of methanol extracts from thirteen Cameroonian edible plants and their antibiotic-potentiating effects against Gram-negative multidrug-resistant (MDR) phenotypes. The broth microdilution method was used to evaluate the minimal inhibitory concentration (MIC) and minimal bactericidal concentration (MBC) of the extracts, as well as their antibiotic-potentiating activities. The phytochemical screening of the extracts was carried out according to the standard methods. The results of phytochemical tests revealed the presence of sterols, polyphenols, and tannins in most of the tested extracts, with the other classes of secondary metabolites being selectively distributed. Tested extracts showed variable antibacterial activities with MIC values ranging from 64 to 1024 *μ*g/mL. However, some extracts were significantly active against certain bacterial strains: seeds extract of* Theobroma cacao* (64 *μ*g/mL) against* Escherichia coli* AG100Atet and* Klebsiella pneumoniae* K24, and the bark extract of* Uapaca guineensis *against* E. coli* ATCC 8739. The leaves extract of* T. cacao* displayed the best MBC values (256 *μ*g/mL) against* E. aerogenes* EA27. Some tested extracts included extracts from the leaves of* T. cacao* and* P. vulgaris, *and the seeds of* D. edulis* and barks* A. indica* has selectively improved (2- to 64-fold) the antibacterial activities of some of the tested antibiotics, chloramphenicol (CHL), tetracycline (TET), kanamycin (KAN), streptomycin (STR), and erythromycin (ERY), against more than 70% of tested MDR bacteria. The findings of this work showed that tested plant extracts and particularly those from* T. cacao* and* Phaseolus vulgaris* can be used alone or in combination with conventional antibiotics in the treatment of infections involving multiresistant bacteria.

## 1. Introduction

The advent of antibiotics has been a tremendous therapeutic progress as it has significantly reduced mortality due to bacterial infections [1]. However, their misuse in humans and animals has contributed to the emergence of drug-resistant bacteria [2]. These bacteria are often more difficult to be combatted, and the infections they caused are more difficult and expensive to be treated. This can therefore lead to serious disability and even death [3, 4]. According to World Health Organization [4], antibiotic resistance has drastically increased to high levels all over the world. The multidrug-resistant (MDR) genes may be carried on the bacterial chromosome, plasmid, or transposons, and their expression allows bacteria to overcome the effects of many antibiotics [5]. Among the antibiotic resistance mechanisms, the most common are enzymatic inactivation of antibiotics, changes in cell permeability, and induction/activation of efflux pumps [6]. The Gram-negative bacteria are among the bacteria that drastically impair the efficacy of antibacterial agents and therefore limit their clinical use [3, 7]. In fact, to guide research, discovery, and development of new antibiotics against MDR bacteria, WHO has developed a global priority list of antibiotic-resistant bacteria where Gram-negative multidrug-resistant (MDR) bacteria, particularly* Pseudomonas aeruginosa* and* Enterobacteriaceae*, constitute the most critical group [4]. These groups of bacteria are particularly characterized by the expression of efflux pumps belonging to the Resistance Nodulation-cell Division (RND) family, which constitutes one of their main resistance mechanisms [8, 9]. Tackling antibiotic resistance mechanisms like efflux pumps expression is therefore a high priority for WHO and scientists over the world. Plant sources used since ancient time to fight microbial infections appear as an interesting alternative for the discovery of new antibacterial substances against MDR bacteria [10–12]. For instance, a number of plant-deriving compounds with possible efflux pump inhibitor (EPI) activities such as reserpine [13], berberine [13, 14], and curcumin [15] have been discovered, but they are still unused clinically. In our previous studies, we have also shown that edible plants could be used alone and/or in combination with commonly used antibiotics to fight infections involving Gram-negative MDR bacteria [16–19]. In the continuous search for natural substances effective in MDR bacteria, this study was aimed at investigating the* in vitro* antibacterial and antibiotic-potentiating activities of methanol extracts of thirteen Cameroonian edible plants (*Azadirachta indica* A. Juss,* Citrus grandis* (L.) (Red),* Citrus grandis* (L.) (White),* Cucurbita maxima* Duch.,* Dacryodes edulis* [G. Don] H. J. Lam,* Hibiscus esculentus* L.,* Ipomoea batatas* (L.) Lam.,* Irvingia gabonensis* (Aubry. Lec. ex O. Rorke) Baill.,* Phaseolus vulgaris* L.,* Saccharum officinarum* L.,* Spondias mombin* L.,* Theobroma cacao* L., and* Uapaca guineensis* Muell. Arg.) against Gram-negative MDR phenotypes. Some of these plants or their related genera are known for their antimicrobial properties, but not as antibiotic modulators (Supplementary Materials, Table S1).

## 2. Material and Methods

### 2.1. Plant Material and Extraction

Plants used in this study were collected in West, Southwest, and Centre regions of Cameroon from March to April 2016. All plants collected were identified at the National Herbarium (Yaoundé, Cameroun) where the voucher specimens were deposited. The names as well as the reference numbers of the studied plants are shown in Table S1 (Supplementary Materials). For the extraction, each plant material was cleaned and air-dried, and the powder (200 g) was soaked in methanol (MeOH, 1 L) for 48 h at room temperature. The extract obtained was collected by filtration using Whatman filter paper n°.1 and concentrated under reduced pressure using a rotary evaporator to yield a residue which constituted the plant extract. All the extracts were then kept at 4°C until further use.

### 2.2. Preliminary Phytochemical Investigations

The major phytochemical classes such as triterpenes (Liebermann-Burchard test), sterols (Salkowski's test), alkaloids (Mayer's test), polyphenols (ferric chloride test), flavonoids (aluminum chloride test), anthraquinones (Borntrager's test), saponins (foam test), and tannins (gelatin test) were investigated as previously described [20, 21].

### 2.3. Chemicals for Antibacterial Assays

Eight reference antibiotics were used in this study: ampicillin (AMP), cefepime (CEF), chloramphenicol (CHL), ciprofloxacin (CIP), erythromycin (ERY), kanamycin (KAN), streptomycin (STP), and tetracycline (TET) which were obtained from Sigma-Aldrich, St Quentin Fallavier, France.* p*-Iodonitrotetrazolium (INT) (Sigma-Aldrich) chloride was used as microbial growth indicator; dimethylsulfoxide (DMSO) was used to dissolve the plant extracts [22].

### 2.4. Bacteria Strains and Culture Media

In this study, we used a panel of 21 strains belonging to Gram-negative bacteria including sensitive and multidrug-resistant strains of* Escherichia coli*,* Enterobacter aerogenes*,* Enterobacter cloacae*,* Klebsiella pneumoniae*,* Providencia stuartii*, and* Pseudomonas aeruginosa*. Their features were previously reported (Supplementary Materials, Table S2). These bacteria were maintained at 4°C and subcultured on a fresh Mueller Hinton Agar (MHA) for 24 h before any antibacterial assay. Mueller Hinton Broth (MHB) was used for antibacterial assays [23].

### 2.5. Antibacterial Assays

MIC and MBC values of the different samples were determined by microdilution using INT colorimetric assay as previously described [22, 24]. Briefly, the samples were dissolved in 10% dimethyl-sulfoxide (DMSO)/Mueller Hinton Broth (MHB) and serially diluted twofold (in a 96-well microplate). Then, 100 *μ*L of inoculum (2 × 10^6^ CFU/mL) prepared in MHB was added in each well. Chloramphenicol was used as reference drug and the well containing the vehicle (DMSO 2.5%) as control. The plates were then covered with a sterile plate sealer and gently shaked to mix the contents of the wells. After 18 h of incubation at 37°C, the MIC value of each sample, defined as the lowest sample concentration that inhibited complete bacteria growth, was detected following addition of 40 *μ*L INT (0.2 mg/mL) and incubation at 37°C for 30 min. Viable bacteria reduced the yellow dye to pink. The MBC value was determined by adding 50 *μ*L aliquots of the preparations, which did not show any growth after incubation during MIC assays, to 150 *μ*L of MHB. Then, these preparations were incubated at 37°C for 48 h. The MBC was regarded as the lowest concentration of samples, which did not produce a color change after addition of INT as mentioned above [24]. Each assay was performed in three independent tests in triplicate. In case there was difference, the MIC or MBC values were taken as the most frequently occurring values.

### 2.6. Antibiotic Resistance Modifying Assay

The resistance modifying activity of the extracts was evaluated by determining the MICs of antibiotics in the presence or absence of the plant extracts in the 96-wells modulation assay as previously described [13, 25]. Briefly, after serial dilutions of antibiotics (256–0.5 *μ*g/mL), the plant extracts were added at their subinhibitory concentrations (MIC/2 and MIC/4) selected after preliminary study assessed against* P. aeruginosa* PA124 (Supplementary Materials, Table S3). The MIC of each treatment was determined as described above. Each assay was performed in three independent tests in duplicate. Modulation factors (MF), calculated as MIC of antibiotic alone/MIC of antibiotic + extract, were used to express the antibiotic-potentiating effects of the plant extracts [11, 26, 27].

## 3. Results

### 3.1. Qualitative Phytochemical Composition of the Tested Extracts

The results of the phytochemical screening (Table 1) showed that only* Theobroma cacao *broad bean (TCBB) extract contained all the classes of screened secondary metabolites. These metabolites were selectively distributed in other tested plant extracts. In addition, results showed that polyphenols, tannins, triterpenes, and steroids were the most represented metabolites in the tested extracts.

### 3.2. Antibacterial Activity of the Tested Extracts

Nineteen extracts from thirteen plants as well as chloramphenicol were tested for their antibacterial activities on a panel of 21 Gram-negative bacteria. The results of Table 2 show that the tested extracts (*A. indica *bark,* P. vulgaris *leaves,* H. esculentus *leaves,* U. guineensis *leaves, and* D. edulis *seed) presented selective antibacterial activity with the recorded MIC values ranging from 64 to 1024 *μ*g/mL. Some extracts presented broad spectrum of antibacterial activity. Their inhibitory activities were observed on 18/21 (85, 71%,* A. indica* bark and* P. vulgaris *leaves), 17/21 (80.95%,* H. esculentus *leaves), and 16/21 (76,19%,* U. guineensis *leaves and* D. edulis *seeds). The lowest MIC value (64 *μ*g/mL) was recorded with the extract of* T. cacao *broad bean (TCBB) against* E. coli* ATCC 8739, AG100A_Tet_, and* K. pneumoniae* K24 and that from bark of* U. guineensis* against* E. coli* ATCC 8739. In general, MBC values were not detected at up to 1024 *μ*g/mL extract concentrations. Extract from* I. batatas* leaves (IBL) and* T. cacao *leaves (TCL) displayed the best MBC values (256 *μ*g/mL) against* E. aerogenes *EA27.

### 3.3. Antibiotic Resistance-Modifying Activities of the Extracts

The antibacterial activity of 6 commonly used antibiotics was evaluated in the presence of plant extracts at the concentrations equivalent to MIC/2 and MIC/4. The results obtained are summarized in Tables 3–9. From these tables, it was observed that some extracts selectively improved the antibacterial activities of tested antibiotics against the selected MDR bacteria (2- to 64-fold decrease of MIC).* D. edulis* seeds extract has significantly improved the antibacterial activities of CHL and KAN against 90% (8/10) and 80% (8/10) of the tested MDR bacteria, respectively (Table 4). The bark extract of* A. indica *(Table 3) and leaves extract of* P. vulgaris* (Table 6) also improved the activities of CHL, STP, and TET to about 80% (8/10) and 70% (7/10) of the tested MDR bacteria, respectively. The modulating effects were also observed after the combination of* T. cacao* leaves extract with STP, CHL, CIP, TET, and STP against 80% to 70% of the tested MDR bacteria (Table 8) whilst other extracts were less active.

## 4. Discussion

Plants constitute an undeniable source of substances named secondary metabolites which are known for their direct or indirect antimicrobial activities; some examples include flavonoids, phenols, terpenoids and sterols, saponins, and tannins [28–30]. The results of the phytochemical screening carried out on the tested extracts indicated the presence of at least one of these metabolites in each of the tested extracts (Table 1). This may explain the antibacterial activities of the extracts tested.

According to Tamokou et al. [31], an edible plant extract is very active if it has a MIC<100 *μ*g/mL, significantly active if 100 ≤ MIC<512 *μ*g/mL, moderately active when 512<MIC ≤ 2048 *μ*g/mL, and weakly active for a MIC>2048 *μ*g/mL. Thus, many of the tested extracts presented significant to moderate activities, 100≤ MIC<2048 *μ*g/mL. Therefore, two extracts were very active (MIC<100 *μ*g/mL), including the extract from seeds of* Theobroma cacao,* active against* E. coli* ATCC8739, AG100Atet, and* K. pneumoniae* K 24 and the extract from bark of* Uapaca guineensis* which was very active on* E. coli* ATCC 8739 (Table 2). Several other studies have already demonstrated the* in vitro* antibacterial activity of at least one of the parts of these two plants or those belonging to the same genus. Previous study has already demonstrated the antibacterial potential of extracts of bark and pulp of* T. cacao* on many bacterial strains including* E. coli* [32]. Singh et al. [33] have also demonstrated the antibacterial activity of* T. cacao* seed extract against* K. pneumoniae*. In addition, the bark of several species of the* Uapaca* genus has shown very good antibacterial activity against certain sensitive and resistant strains [34, 35]; the results obtained in this work reinforce those previous works done with some of the tested extracts. The extracts of seeds of* Theobroma cacao *and that from bark of* Uapaca guineensis* could be used to fight infections involving multidrug-resistant bacteria.

In addition to their direct antibacterial activities, secondary metabolites have been found to act indirectly as modulators of the activity of antibacterial agents [10, 28, 36]. In this work, some antibiotics (CHL, TET, KAN, STR, and ERY) activities were improved (2 to 64 times) on more than 70% of the multidrug-resistant bacteria tested in the presence of* T. cacao *leaves,* P. vulgaris* leaves,* D. edulis* seeds, and* A. indica* barks extracts (Tables 3–9). The bacteria used in this work are multiresistant and overexpress efflux pumps as a resistance mechanism (Supplementary Materials, Table S2). This suggests that aforesaid extracts could contain substances which are able to inhibit the efflux pumps expressed in these bacteria [37], thus leading to an increase in the effectiveness of antibiotics [38]. Several studies have shown that polyphenols, especially flavonoids, could improve the activity of antibiotics against resistant bacterial strains [39, 40]. Thus, the presence of these metabolites in the most active extracts may be the origin of the observed antibiotic-potentiating activity. Many cases of antagonism were also observed and this could be due to the negative interactions between the antibiotics and the compounds of the plant extract, leading, for example, to the inhibition of the active groups of the antibiotics. These results of this study indicate, for the first time, the potential of the tested plant extracts, mainly extracts from* D. edulis*,* P. vulgaris, A. indica*, and* T. cacao*, to reverse antibiotic resistance.

## 5. Conclusion

This work has provided informative data related to the antimicrobial activity of the tested plant extracts. It suggests that plant extracts and particularly those from* Theobroma cacao* and* P. vulgaris* can be used alone or in combination with conventional antibiotics in the treatment of bacterial infections involving multiresistant phenotypes.

## Figures and Tables

**Table 1:** Extraction yields and phytochemical composition of the plant extracts.

**Plant extract**	**Part used**	**Yields (%)**	**ALK**	**POL**	**FLAV**	**ANTHR**	**TAN**	**TRI**	**STER**	**SAP**
***Azadirachta indica***	Bark	10.3	**+**	**+**	-	-	**+**	-	**+**	**+**
***Citrus grandis *(Red)**	Pericarp	13.4	**+**	**+**	**+**	-	**+**	**+**	**+**	-
	Leaves	6.2	-	-	-	-	-	**+**	**+**	-
***Citrus grandis *(White)**	Leaves	2.6	**+**	**+**	-	-	**+**	**+**	**+**	-
***Cucurbita maxima***	Beans	2.6	-	**+**	-	-	**+**	**+**	**+**	**+**
***Dacryodes edulis***	Leaves	6.2	-	**+**	**+**	**+**	**+**	**+**	**+**	**+**
	Bark	9.1	-	**+**	-	**+**	**+**	**+**	**+**	**+**
	Seeds	6.9	-	**+**	**+**	**+**	**+**	**+**	**+**	**+**
***Hibiscus esculentus***	Leaves	1.9	-	**+**	-	-	**+**	-	**+**	-
***Ipomoea batatas***	Beans	3.3	**+**	**+**	**+**	**+**	**+**	**+**	-	**+**
***Irvingia gabonensis***	Leaves	6.7	-	**+**	-	-	**+**	-	**+**	**+**
***Phaseolus vulgaris***	Leaves	1.2	-	**+**	-	-	**+**	-	**+**	**+**
***Spondias mombin***	Leaves	21.4	-	**+**	-	-	**+**	**+**	**+**	-
***Saccharum officinarum***	Leaves	8.4	-	**+**	-	-	**+**	-	**+**	**+**
***Theobroma cacao***	Leaves	3.1	-	**+**	-	-	**+**	**+**	**+**	**+**
	Beans	6.2	**+**	**+**	**+**	**+**	**+**	**+**	**+**	**+**
***Uapaca guineensis***	Leaves	7.3	-	**+**	-	-	**+**	**+**	**+**	**+**
	Bark	6.1	**+**	**+**	-	-	**+**	**+**	**+**	**+**

(-): absent; (+): present; yield calculated as the ratio of the mass of the obtained methanol extract/mass of the plant powder; ALK: alkaloids; ANTH: anthocyanins; ANTHR: anthraquinones; FLAV: flavonoids; POL: polyphenols; SAP: saponins; STER: steroids; TAN: tannins; TRI: triterpenes.

**Table 2:** MIC and MBC of the plant extracts and chloramphenicol against bacteria strain.

**Bacterial strains** ^**a**^	**Samples** ^**b**^ ** MIC and MBC in *μ*g/mL (in Bracket)**																		
	**Plant extracts**																		**Antibiotic**
	**AIB**	**CGrP**	**CGrL**	**CGwL**	**CMB**	**DEB**	**DEL**	**DES**	**HEL**	**IBL**	**IGB**	**PVL**	**SML**	**SOL**	**TCBB**	**TCL**	**UGB**	**UGL**	**CHL**
***E. coli***																			
**ATTC8739**	1024 (-)	1024 (-)	1024 (-)	1024 (-)	1024 (-)	-	-	1024 (-)	512 (-)	1024 (-)	-	512 (-)	-	1024 (-)	**64 (-)**	1024 **(-)**	**64** (1024)	256(1024)	8 (64)
**ATCC10536**	1024 (-)	1024 (-)	1024 (-)	-	1024 (-)	-	-	-	1024 (-)	-	-	512 (-)	-	-	-	-	128 (-)	1024 (-)	4 (16)
**AG100**	1024 (-)	-	1024 (-)	512 (-)	1024 (-)	-	1024 (-)	-	512 (-)	-	1024 (-)	512 (1024)	-	1024 (-)	1024 **(-)**	-	512 (-)	512 (-)	32 (64)
**AG102**	1024 (-)	-	512 (-)	-	1024 (-)	-	-	-	1024 (-)	-	-	512 (-)	-	512 (-)	256 **(-)**	-	256 (1024)	-	32 (256)
**AG100Atet**	1024 (-)	1024 (-)	256 (-)	512 (-)	-	-	1024 (-)	-	1024 (-)	1024 (-)	-	-	-	-	**64 (-)**	1024 **(-)**	256 (-)	512 (-)	4 (32)
**MC4100**	1024 (-)	-	256 (-)	-	128 (1024)	-	1024 (-)	1024 (-)	512 (-)	-	-	256 (512)	-	1024 (-)	-	-	-	512 (-)	128 (-)
**W3110**	1024 (-)	-	1024 (-)	-	-	1024 (-)	-	1024 (-)	512 (1024)	-	-	1024 (-)	-	1024 (-)	-	-	-	512 (-)	8 (32)
***E. aerogenes***																			
**ATCC13048**	1024 (-)	-	-	-	-	1024 (-)	512 (-)	512 (-)	256 (-)	256 (-)	-	1024 (-)	-	512 (-)	-	256 **(-)**	-	512 (-)	8 (128)
**EA27**	512 (1024)	-	-	1024 (-)	-	1024 (-)	512 (1024)	1024 (-)	512 (1024)	256 (256)	-	512 (1024)	-	512 (-)	256 **(-)**	256 (256**)**	1024 (-)	1024 (-)	128 (256)
**EA289**	1024 (-)	-	1024 (-)	128(-)	512 (-)	512 (-)	256 (1024**)**	512 (-)	512 (-)	1024 (-)	-	1024 (-)	1024 (-)	1024 (-)	1024 **(-)**	1024 **(-)**	-	-	4 (64)
**EA294**	1024 (-)	-	1024 (-)	-	-	1024 (-)	512 (-)	128 (512)	-	-	-	512 (-)	-	-	-	-	-	512 (-)	2 (256)
**EA 298**	512 (-)	1024 (-)	-	1024 (-)	-	-	-	1024 (-)	256 (-)	512 (-)	-	1024 (-)	1024 (-)	512 (-)	-	512 **(-)**	1024 (-)	1024 (-)	8 (128)
***K. pneumoniae***											-								
**ATCC11296**	-	1024 (-)	1024 (-)	128(-)	-	-	-	1024 (-)	512 (-)	512 (-)	1024 (-)	512 (-)	-	-	-	512 **(-)**	-	1024 (-)	8 (256)
**K24**	1024 (-)	-	128 (512)	-	-	1024 (-)	512 (-)	-	512 (-)	-	-	512 (-)	-	1024 (-)	**64 (-)**	-	128 (-)	512 (-)	16 (128)
**KP55**	1024 (-)	-	-	-	128 (1024)	-	-	1024 (-)	512 (-)	512 (-)	-	-	1024 (-)	512 (-)	1024 **(-)**	512 **(-)**	512 (-)	1024 (-)	64 (128)
**KP63**	512 (1024)	-	-	-	-	1024 (-)	-	512 (-)	-	256 (-)	-	512 (-)	-	-	1024 **(-)**	256 **(-)**	-	-	16(128)
***P. stuartii***																			
**PS2636**	-	-	-	-	-	512 (-)	512 (-)	512 (-)	512 (1024)	-	-	512 (-)	-	-	-	-	-	1024 (-)	64 (256)
**NEA16**	-	-	-	-	-	512 (-)	512 (-)	256 (-)	512 (-)	-	-	-	-	1024 (-)	-	-	-	1024 (-)	64 (128)
***E. cloacae***																			
**ECCI69**	512 (-)	1024 (-)	-	-	-	128 (-)	256 (1024)	128 (-)	-	512 (-)	-	512 (-)	-	-	1024 **(-)**	512 **(-)**	-	512 (-)	128 (-)
***P. aeruginosa***																			
**PA01**	1024 (-)	-	1024 (-)	-	-	512 (-)	1024 (-)	512 (1024)	-	-	-	512 (1024)	-	1024 (-)	1024 **(-)**	-	128 (-)	-	128 (-)
**PA124**	1024 (-)	1024(-)	512 (-)	512 (-)	-	-	-	1024 (-)	512(-)	1024 (-)	-	512 (-)	-	1024 (-)	-	-	512 (-)	-	32 (-)

^a^Bacterial strain [*E.c*: *Escherichia coli*, *E.a*: *Enterobacter aerogenes*, *K.p*: *Klebsiella pneumoniae*, *P.s*: *Providencia stuartii*, *E.cl*: *Enterobacter cloacae*, *P.a*: *Pseudomonas aeruginosa*]. ^b^Samples [AIB: *Azadirachta indica* bark, CGrP: *Citrus grandis* (red) pericarp, CGrL: *Citrus grandis* (red) leaves, CGwL: *Citrus grandis* (white) leaves, CMB: *Cucurbita maxima* beans, DEB: *Dacryodes edulis* bark, DEL: *Dacryodes edulis* leaves, DES: *Dacryodes edulis* seeds, HEL: *Hibiscus esculentus* leaves, IBL: *Ipomoea batatas* leaves, IGB: *Irvingia gabonensis* beans, PVL: *Phaseolus vulgaris* leaves, SML: *Spondias mombin* leaves, SOL: *Saccharum officinarum* leaves, TCBB: *Theobroma cacao* beans, TCL: *Theobroma cacao* leaves, UGB: *Uapaca guineensis* bark, UGL: *Uapaca guineensis* leaves, CHL: chloramphenicol]. MIC: minimal inhibitory concentration, MBC: minimal bactericidal concentration, -: MIC and MBC at up to 1024 *μ*g/mL; MIC in bold: significant activity.

**Table 3:** Antibiotic resistance modulatory activity of leaves extract of *Azadirachta indica*.

**Antibiotics**	**Extract concentration**	**Bacteria MIC (**μ**g/mL) and modulating factors (in bracket)**										
		*E. coli*		*E. aerogenes*		*K. pneumoniae*		*P. stuartii*		*P.aeruginosa*		Modulating effect (%)
		AG102	AG100Atet	EA27	EA289	KP55	KP63	PS2636	NEA16	PA01	PA124	
**CHL**	0	64	8	64	64	64	64	32	64	64	32	
	CMI/2	16 (**4**)	8 (1)	32 (**2**)	64 (1)	8 (**8**)	32 (**2**)	1 (**16**)	16 (**4**)	32 (**2**)	8 **(4**)	**80.00**
	CMI/4	32 (**2**)	8 (1)	32 (**2**)	64 (1)	64 (1)	32 (**2**)	4 (**8**)	32 (**2**)	32 (**2**)	8 **(4**)	**70.00**
**KAN**	0	32	4	16	32	64	64	16	32	16	64	
	CMI/2	16 (**2**)	4 (1)	16 (1)	32(1)	64 (1)	64 (1)	16 (1)	4 (**4**)	4 (**4**)	16 **(4**)	40.00
	CMI/4	16 (**2**)	4 (1)	16 (1)	32(1)	64 (**1**)	64 (1)	16 (1)	4 (**8**)	16(1)	32 (**2)**	40.00
**STP**	0	128	256	256	64	64	256	256	16	256	64	
	CMI/2	128(1)	256(1)	256(1)	**32 (2)**	**8 (8)**	**32 (8)**	**128 (2)**	16(1)	256(1)	32 (**2)**	50.00
	CMI/4	128(1)	256(1)	256(1)	64 (1)	128 (2)	**32(8)**	256 (1)	32 (2)	256(1)	32 (**2)**	20.00
**CIP**	0	8	1	1	1	8	4	16	2	2	16	
	CMI/2	8(1)	1 (1)	1 (1)	1 (1)	**<0.5(≥16)**	4 (1)	16 (1)	**<0.5(>4)**	1(**2**)	8 **(2**)	20.00
	CMI/4	8(1)	2(1)	1 (1)	1(1)	16 (1)	4 (1)	16 (1)	2 (1)	2(1)	16(1)	00.00
**TET**	0	8	< 0.5	64	32	16	32	4	32	16	16	
	CMI/2	1(**8**)	< 0.5(na)	64 (1)	8(**4**)	2(**8**)	16 (**2**)	1 (**4**)	16 (**2**)	2 (**8**)	2 (**8**)	**80.00**
	CMI/4	4 (**2**)	< 0.5(na)	64 (1)	8(**4**)	4(**4**)	16 (**2**)	1 (**4**)	32 (1)	2 (**8**)	2 (**8**)	**70.00**
**ERY**	0	64	8	16	64	64	32	16	32	16	32	
	CMI/2	32 (**2**)	2 (**4**)	16 (1)	32 (**2**)	4 (**32**)	32 (1)	2 (**8**)	32 (1)	8 (**2**)	16 (**4)**	**70.00**
	CMI/4	32 (**2**)	4 (**2**)	16 (1)	64 (1)	32 (**2**)	32 (1)	4 (**4**)	32 (1)	16 (1)	32(1)	40.00

CHL: chloramphenicol, KAN: kanamycin, STP: streptomycin, CIP: ciprofloxacin, TET: tetracycline, ERY: erythromycin; −: MIC not detected at up to 256 *μ*g/mL; ( ): modulating factor; MIC: minimal inhibitory concentration; values in bold represent modulating factor ≥ 2.

**Table 4:** Antibiotic resistance modulatory activity of seeds extract of* Dacryodes edulis*.

**Antibiotics**	**Extract concentration**	**Bacteria MIC (**μ**g/mL) and modulating factors (in bracket)**										
		*E. coli*		*E. aerogenes*		*K. pneumoniae*		*P. stuartii*		*P. aeruginosa*		Modulating effect (%)
		AG102	AG100Atet	EA27	EA289	KP55	KP63	PS2636	NEA16	PA01	PA124	
**CHL**	0	64	8	64	64	64	64	32	64	64	32	
	CMI/2	<2(**≥32**)	2 (**4**)	32 **(2**)	64 (1)	16 (**4**)	16 (**4**)	16 (**2**)	16 (**4**)	32 (**2**)	4 (**8**)	**90.00**
	CMI/4	8 **(8**)	2 (**4**)	32 (**2**)	64 (1)	32 **(2**)	64 (1)	16 (**2)**	32 (**2**)	64 (1)	4 (**8**)	**70.00**
**KAN**	0	32	4	16	32	64	64	16	32	16	64	
	CMI/2	8 (**4)**	2 (**2**)	8 (**2**)	32(1)	64 (1)	32 (**2**)	2 (**8**)	4 (**8**)	8 (**2**)	1 (**64**)	**80.00**
	CMI/4	16 (**2**)	2 (**2**)	16 (0.5)	32(1)	64 (1)	32 (**2**)	2 (**8**)	8 (**4**)	16 (1)	32 (**2**)	60.00
**STP**	0	128	256	256	64	64	256	256	16	256	64	
	CMI/2	64 (**2**)	128(**2**)	256(1)	64(1)	128(0.5)	256(1)	256(1)	32 (**2**)	256(1)	1 (**64**)	40.00
	CMI/4	64 (**2**)	256(1)	256(1)	64(1)	128(0.5)	256(1)	256(1)	32 (**2**)	256(1)	32 (**2**)	20.00
**CIP**	0	8	1	1	1	8	4	16	2	2	16	
	CMI/2	8(1)	0.5 (**2**)	2 (0.5)	1(1)	2(**4**)	2 (**2**)	4 (**4)**	2 (1)	1 (**4**)	1 (**16**)	60.00
	CMI/4	8(1)	2 (2)	2 (0.5)	1(1)	2(**4**)	2 (**2**)	16 (1)	2 (1)	2 (1)	4 (**4**)	30.00
**TET**	0	8	< 0,5	64	32	16	32	4	32	16	16	
	CMI/2	2 (**8**)	< 0,5 (na)	-(na)	8(**4**)	1(**16**)	16 **(2**)	1 (**4**)	32 (1)	2 (**8**)	1 (**16**)	**70.00**
	CMI/4	2 (**8**)	< 0,5 (na)	- (na)	8(**4**)	2(**8**)	16 (**2**)	1 (**4**)	32 (1)	2 (**8**)	4 (**4**)	**70.00**
**ERY**	0	64	8	16	64	64	32	16	32	16	32	
	CMI/2	16 (**4**)	2 (**4**)	16 (1)	64 (1)	16 (**4**)	32 (1)	2 (**8**)	64 (0.5)	8 (**2**)	**8 (4)**	60.00
	CMI/4	16(**4**)	2 (**4**)	16 (1)	64 (1)	32 (**2**)	32 (1)	16 (1)	64 (0.5)	8 (**2**)	32 (1)	40.00

CHL: chloramphenicol, KAN: kanamycin, STP: streptomycin, CIP: ciprofloxacin, TET: tetracycline, ERY: erythromycin; −: MIC not detected at up to 256 *μ*g/mL; ( ): modulating factor; MIC: minimal inhibitory concentration; values in bold represent modulating factor ≥ 2.

**Table 5:** Antibiotic resistance modulatory activity of leaves extract of *Ipomea batatas*.

**Antibiotics**	**Extract concentration**	**Bacteria MIC (**μ**g/mL) and modulating factors (in bracket)**										
		*E. coli*		*E. aerogenes*		*K. pneumoniae*		*P. stuartii*		*P. aeruginosa*		Modulating effect (%)
		AG102	AG100Atet	EA27	EA289	KP55	KP63	PS2636	NEA16	PA01	PA124	
**CHL**	0	64	8	64	64	64	64	32	64	64	32	
	CMI/2	32 (**2**)	8 (1)	16 (**4**)	64 (1)	64 (1)	32 (**2**)	32 (1)	32 (**2**)	32 (**2**)	16 **(2)**	60.00
	CMI/4	32 (**2**)	8 (1)	32 (**2**)	64 (1)	64 (1)	16 (**4**)	32 (1)	32 (**2**)	32 (**2**)	16 **(2)**	60.00
**KAN**	0	32	4	16	32	64	64	8	32	16	64	
	CMI/2	32 (1)	4 (1)	8 (**2**)	32 (1)	64 (1)	32 (**2**)	4 (**2**)	16 (**2**)	8 (**2**)	16(**4**)	60.00
	CMI/4	32 (1)	4 (1)	8 (**2**)	32 (1)	64 (1)	32 (**2**)	4 (**2**)	16 (**2**)	8 (**2**)	32 (**2**)	60.00
**STP**	0	128	256	256	64	64	256	>256	16	256	64	
	CMI/2	64 (**2**)	256 (1)	64 (**4**)	64 (1)	32(**2**)	64 (**4**)	128 (**≥2**)	32(**2**)	64(**4**)	64** (**1)	**70.00**
	CMI/4	64 (**2**)	256 (1)	64 (**4**)	128(1)	64(1)	64 (**4**)	256 (**≥2**)	32(**2**)	64(**4**)	64** (**1)	60.00
**CIP**	0	8	1	1	1	8	1	4	2	2	16	
	CMI/2	2 (**4**)	0.5 (**2**)	1 (1)	1(1)	8 (1)	<0.5(**≥2**)	1 (**4**)	2 (1)	<0.5(**≥4**)	16(1)	40.00
	CMI/4	2 (**4**)	0.5 (**2**)	1 (1)	1 (1)	8 (1)	<0.5(**≥4**)	2 (**2**)	4 (1)	<0.5(**≥4**)	16(1)	50.00
**TET**	0	8	< 0.5	64	32	16	32	4	32	16	16	
	CMI/2	8 (1)	< 0.5 (na)	64 (1)	8 (**4**)	16 (1)	32(1)	2 (**2**)	4 (**8**)	4 (**4**)	8(**2**)	50.00
	CMI/4	8 (1)	< 0.5 (na)	64 (1)	8 (**4**)	16 (1)	16 (**2**)	2 (**2**)	16 (**2**)	4 (**4**)	8(**2**)	60.00
**ERY**	0	64	8	16	64	64	32	16	32	16	32	
	CMI/2	16 (**4**)	8 (1)	8 (**2**)	64 (1)	16 (**4**)	16 (**2**)	2 (**8**)	8 (**4**)	16 (1)	32 (1)	60.00
	CMI/4	16 (**4**)	8 (1)	8 (**2**)	64 (1)	32 (**2)**	16 (**2**)	4(**4**)	8 (**4**)	16 (1)	32 (1)	60.00

CHL: chloramphenicol, KAN: kanamycin, STP: streptomycin, CIP: ciprofloxacin, TET: tetracycline, ERY: erythromycin; −: MIC not detected at up to 256 *μ*g/mL; ( ): modulating factor; MIC: minimal inhibitory concentration; values in bold represent modulating factor ≥ 2.

**Table 6:** Antibiotic resistance modulatory activity of leaves extract of *Phaseolus vulgaris*.

**Antibiotics**	**Extract concentration**	**Bacteria MIC (**μ**g/mL) and modulating factors (in bracket)**										
		*E. coli*		*E. aerogenes*		*K. pneumoniae*		*P. stuartii*		*P. aeruginosa*		Modulating effect (%)
		AG102	AG100Atet	EA27	EA289	KP55	KP63	PS2636	NEA16	PA01	PA124	
**CHL**	0	64	8	64	64	64	64	32	64	64	32	
	CMI/2	32 (**2**)	2 (**4**)	64(1)	32 (**2**)	32 (**2**)	32 (**2**)	8 (**4**)	32 (**2**)	64 (1)	16 **(2)**	**80.00**
	CMI/4	32 (**2**)	4 (**2**)	64(1)	64 (1)	32(**2**)	32 (**2**)	8 (**4**)	32 (**2**)	64 (1)	16 **(2)**	**70.00**
**KAN**	0	32	4	16	32	64	64	8	32	16	64	
	CMI/2	8 (**4**)	4 (1)	8 (**2**)	32(1)	64 (1)	16 (**4**)	2 (**4**)	16(**4**)	16 (1)	16(**4**)	60.00
	CMI/4	16 (**2**)	4 (1)	8 (**2)**	32(1)	64(1)	32 (**2**)	2 (**4**)	16 (**2**)	16 (1)	32 (**2**)	60.00
**STP**	0	128	256	256	64	64	256	>256	16	256	64	
	CMI/2	64 (**2**)	128 (**2**)	16 (**16)**	32 (**2**)	8 (**8**)	4(**64**)	256(**≥2**)	16(1)	256(1)	64** (**1)	**70.00**
	CMI/4	64 (**2**)	256(1)	64 (**4**)	64(1)	32 (**2**)	8(**32**)	256(1)	16(1)	256(1)	64** (**1)	40.00
**CIP**	0	8	1	1	1	8	1	4	2	2	16	
	CMI/2	8(1)	<0.5 (**≥2**)	1(1)	1(1)	4(**2**)	1 (1)	4 (1)	2 (1)	1 (**2**)	16(1)	30.00
	CMI/4	8(1)	<0.5(<0.5)	1 (1)	1(1)	4(**2**)	1 (1)	4 (1)	2 (1)	2 (1)	16(1)	10.00
**TET**	0	8	< 0.5	64	32	16	32	4	32	16	16	
	CMI/2	0.5 (**16**)	< 0.5 (na)	64 (1)	16 (**2**)	2(**8**)	8 (**4**)	1 (**4**)	32 (1)	2 (**8**)	8(**2**)	**70.00**
	CMI/4	4 (**2**)	< 0.5 (na)	64 (1)	16 (**2**)	8(**2**)	8 (**4**)	1 (**4**)	32 (1)	2 (**8**)	8(**2**)	**70.00**
**ERY**	0	64	8	16	64	64	32	16	32	16	32	
	CMI/2	16(**4**)	2 (**4**)	16 (1)	32 (**2**)	32 (**2**)	16 (**2**)	4 (**4**)	32 (1)	16 (1)	32 (1)	60.00
	CMI/4	16(**4**)	2 (**4**)	16 (1)	32 (**2**)	32 (**2**)	16 (**2**)	8 (**2**)	32 (1)	16 (1)	32 (1)	60.00

CHL: chloramphenicol, KAN: kanamycin, STP: streptomycin, CIP: ciprofloxacin, TET: tetracycline, ERY: erythromycin; −: MIC not detected at up to 256 *μ*g/mL; ( ): modulating factor; MIC: minimal inhibitory concentration; values in bold represent modulating factor ≥ 2.

**Table 7:** Antibiotic resistance modulatory activity of broad bean extract of *Theobroma cacao*.

**Antibiotics**	**Extract concentration**	**Bacteria MIC (**μ**g/mL) and modulating factors (in bracket)**										
		*E. coli*		*E. aerogenes*		*K. pneumoniae*		*P. stuartii*		*P. aeruginosa*		Modulating effect (%)
		AG102	AG100Atet	EA27	EA289	KP55	KP63	PS2636	NEA16	PA01	PA124	
**CHL**		64	8	64	64	64	64	32	64	64	32	
	CMI/2	32 (**2**)	8 (1)	32 (**2**)	128 (0.5)	128 (0.5)	16 (**4**)	8 (**4**)	16 (**4**)	64 (1)	32 (1)	50.00
	CMI/4	32 (**2**)	8 (1)	32 (**2**)	128 (0.5)	128 (0.5)	64 (1)	8 (**4**)	16 (**4**)	64 (1)	32 (1)	40.00
**KAN**	0	32	4	16	32	64	64	8	32	16	64	
	CMI/2	16 (**2**)	4 (1)	8 (**2**)	64 (1)	64 (1)	64 (1)	2 (**4**)	8 (**4**)	16 (1)	32 (**2**)	50.00
	CMI/4	16 (**2**)	4 (1)	8 (**2**)	64 (1)	64 (1)	64 (1)	4 (**2**)	16 (**2**)	16 (1)	32 (**2**)	50.00
**STP**	0	128	256	256	64	64	256	>256	16	256	64	
	CMI/2	128 (1)	- (na)	256 (1)	64 (1)	32(**2**)	128(**2**)	32(**<8**)	16 (1)	256 (1)	32 (**2**)	40.00
	CMI/4	128 (1)	- (na)	256 (1)	128(**2**)	32(**2**)	256(1)	16(**<16**)	16(1)	256 (1)	32 (**2**)	40.00
**CIP**	0	8	1	1	1	8	1	4	2	2	16	
	CMI/2	4 (**2**)	1(1)	1 (1)	0.5 (**2**)	16 (0.5)	0.5 (**2**)	<0.5(**<8**)	2 (1)	0.5 (**4**)	8(**2**)	60.00
	CMI/4	4 (**2**)	1(1)	2 (1)	1(1)	16 (0.5)	0.5 (**2**)	<0.5(**<8**)	4 (0.5)	1 (2)	8(**2**)	40.00
**TET**	0	8	< 0.5	64	32	16	32	4	32	16	16	
	CMI/2	8(1)	< 0.5 (na)	64 (1)	16 (**2**)	16 (**2**)	8(**4**)	0.5 (**8**)	4 (**8**)	8 (**2**)	8(**2**)	**70.00**
	CMI/4	8(1)	< 0.5 (na)	64 (1)	32 (1)	16(1)	16 (**2**)	0.5 (**8**)	4 (**8**)	4 (**4**)	8(**2**)	50.00
**ERY**	0	64	8	16	64	64	32	16	32	16	*32*	
	CMI/2	32 (**2**)	8 (1)	8 (**2**)	128 (0.5)	16 (**4**)	8 (**4**)	16 (1)	4 (**8**)	16 (1)	16 (**2**)	60.00
	CMI/4	32 (**2**)	8 (1)	16 (1)	128 (0.5)	32(**2**)	16 (**2**)	16 (1)	16 (**2**)	16 (1)	16 (**2**)	50.00

CHL: chloramphenicol, KAN: kanamycin, STP: streptomycin, CIP: ciprofloxacin, TET: tetracycline, ERY: erythromycin; −: MIC not detected at up to 256 *μ*g/mL; ( ): modulating factor; MIC: minimal inhibitory concentration; values in bold represent modulating factor ≥ 2.

**Table 8:** Antibiotic resistance modulatory activity of leaves extract of *Theobroma cacao*.

**Antibiotics**	**Extract concentration**	**Bacteria MIC (**μ**g/mL) and modulating factors (in bracket)**										
		*E. coli*		*E. aerogenes*		*K. pneumoniae*		*P. stuartii*		*P. aeruginosa*		Modulating effect (%)
		AG102	AG100Atet	EA27	EA289	KP55	KP63	PS2636	NEA16	PA01	PA124	
**CHL**	0	64	8	64	64	64	64	32	64	32	64	
	CMI/2	32 (**2**)	2 (**4**)	32 (**2**)	16 (**4**)	16 (**4**)	32 (**2**)	32 (1)	16 (**4**)	32 (1)	64 (1)	**70.00**
	CMI/4	32 (**2**)	2 (**4**)	64 (1)	64 (1)	32 (**2**)	32 (**2**)	8 (**4**)	32 (**2**)	32 (1)	64 (1)	60.00
**KAN**	0	32	4	16	32	64	64	8	32	64	16	
	CMI/2	32(1)	4 (1)	8 (**2**)	16 (**2**)	32 (**2**)	16 (**4**)	< 2 (**≥4**)	16 (**4**)	64 (1)	16 (1)	60.00
	CMI/4	32 (1)	4 (1)	32 (**2**)	16 (**2**)	64 (1)	16 (**4**)	2 (**4**)	16 (**2**)	64 (1)	16 (1)	50.00
**STP**	0	128	256	256	64	64	256	256	16	32	256	
	CMI/2	64 (**2**)	128 (**2**)	128 (**2**)	64 (1)	32(**2**)	32(**4**)	8 (**32**)	32 (1)	8 (**4**)	128 (**2**)	**80.00**
	CMI/4	128 (1)	256(1)	**128 (2)**	64 (1)	64 (1)	32**(4)**	128** (2)**	32(1)	16 **(2**)	128 **(2**)	50.00
**CIP**	0	8	1	1	1	8	1	4	2	16	2	
	CMI/2	4 (**2**)	1 (1)	1 (1)	0.5 (**2**)	8 (1)	<0.5(**≥2**)	0.5(**8**)	0.5 (**4**)	8 (**2**)	0.5** (4)**	**70.00**
	CMI/4	8 (1)	1 (1)	4 (0.25)	2 (2)	16 (2)	**<**0.5**(≥2)**	4 (1)	2 (1)	8 (**2**)	0.5** (4)**	30.00
**TET**	0	8	< 0.5	64	32	16	32	4	32	16	16	
	CMI/2	4 (**2**)	< 0.5 (na)	32 (**2**)	16 (**2**)	16(1)	16 (**2**)	4 (1)	4 (**8**)	8 (**2**)	4 (**4**)	**70.00**
	CMI/4	8(1)	< 0.5 (na)	32 (**2**)	16 (**2**)	16(1)	16 (**2**)	8 (**2**)	16 (**2**)	8 (**2**)	4 (**4**)	**70.00**
**ERY**	0	64	8	16	64	64	32	16	32	32	16	
	CMI/2	32 (**2**)	8 (1)	8 (**2**)	128 (1)	32(**2**)	8 (**2**)	16 (1)	8 (**4**)	8 (**4**)	**8 (2)**	**70.00**
	CMI/4	32 (**2**)	8 (1)	8 (**2**)	64 (1)	32(**2**)	16 (**4**)	16 (1)	8 (**4**)	16 (**2**)	16(1)	60.00

CHL: chloramphenicol, KAN: kanamycin, STP: streptomycin, CIP: ciprofloxacin, TET: tetracycline, ERY: erythromycin; −: MIC not detected at up to 256 *μ*g/mL; ( ): modulating factor; MIC: minimal inhibitory concentration; values in bold represent modulating factor ≥ 2.

**Table 9:** Antibiotic resistance modulatory activity of barks extract of *Uapaca guineensis*.

**Antibiotics**	**Extract concentration**	**Bacteria MIC (**μ**g/mL) and modulating factors (in bracket)**										
		*E. coli*		*E. aerogenes*		*K. pneumoniae*		*P. stuartii*		*P. aeruginosa*		Modulating effect (%)
		AG102	AG100Atet	EA27	EA289	KP55	KP63	PS2636	NEA16	PA01	PA124	
**CHL**	0	64	8	64	64	64	64	32	64	64	32	
	CMI/2	32 (**2**)	8 (1)	32 (**2**)	128(0.5)	64 (1)	16 (**4**)	8 (**4**)	16 (**4**)	64 (1)	32 (1)	50.00
	CMI/4	32 (**2**)	8 (1)	32 (**2**)	128(0.5)	64 (1)	64 (1)	8 (**4**)	16 (**4**)	64 (1)	32 (1)	40.00
**KAN**	0	32	4	16	32	64	64	8	32	16	64	
	CMI/2	16 (**2**)	4 (1)	8 (**2**)	64 (**2**)	64 (1)	128 (1)	2 (**4**)	8 (**4**)	16 (1)	32 (**2**)	60.00
	CMI/4	16 (**2**)	4 (1)	8 (**2**)	64 (**2**)	64 (1)	64 (1)	4 (**2**)	16 (**2**)	16 (1)	32 (**2**)	60.00
**STP**	0	128	256	256	64	64	256	>256	16	256	64	
	CMI/2	128 (1)	- (na)	256 (1)	64 (1)	32(**2**)	128 (**2**)	32(**≥8**)	16 (1)	256 (1)	32 (**2**)	40.00
	CMI/4	128 (1)	- (na)	256 (1)	128(**2**)	32(**2**)	256(1)	16(**≥8**)	16 (1)	256 (1)	32 (**2**)	40.00
**CIP**	0	8	1	1	1	8	1	4	2	2	16	
	CMI/2	4 (**2**)	1(1)	1 (1)	0.5 (**2**)	16 (**2**)	0.5 (**2**)	<0.5 (**≥8)**	2 (1)	0.5 (**4**)	8(**2**)	**70.00**
	CMI/4	4 (**2**)	1(1)	1 (1)	1(1)	16 (**2**)	0.5 (**2**)	<0.5 (**≥8)**	2 (1)	1 (**2**)	8(**2**)	60.00
**TET**	0	8	< 0.5	64	32	16	32	4	32	16	16	
	CMI/2	8(1)	<0.5 (na)	64 (1)	16 (**2**)	16 (**2**)	8(**4**)	0.5 (**8**)	4 (**8**)	8 (**2**)	8(**2**)	**70.00**
	CMI/4	8(1)	<0.5 (na)	64 (1)	32 (1)	16(1)	16 (**2**)	0.5 (**8**)	4 (**8**)	4 (**4**)	8(**2**)	50.00
**ERY**	0	64	8	16	64	64	32	16	32	16	32	
	CMI/2	32 (**2**)	8 (1)	8 (**2**)	64 (1)	16 (**4**)	8 (**4**)	16 (1)	4 (**8**)	16 (1)	16 (**2**)	60.00
	CMI/4	32 (**2**)	8 (1)	16 (1)	64 (1)	32(**2**)	16 (**2**)	16 (1)	16 (**2**)	16 (1)	16 (**2**)	50.00

CHL: chloramphenicol, KAN: kanamycin, STP: streptomycin, CIP: ciprofloxacin, TET: tetracycline, ERY: erythromycin; −: MIC not detected at up to 256 *μ*g/mL; ( ): modulating factor; MIC: minimal inhibitory concentration; values in bold represent modulating factor ≥ 2.

## Data Availability

The data used to support the findings of this study are included within the article.

## Supplementary Materials

Supplementary Materials Supplementary file.docx. Table S1: information on the studied plants; Table S2: further details on the antibiotic resistance profiles of tested Gram-negative bacteria; Table S3: results of preliminary evaluation of antibiotic resistance modulatory activity of selected extracts at subinhibitory concentrations against* Pseudomonas aeruginosa* PA124.
