# Supplementary material for: Antibacterial and Antibiotic-Potentiating Activities of Thirteen Cameroonian Edible Plants against Gram-Negative Resistant Phenotypes
Source: ScientificWorldJournal. 2018 Sep 10;2018:4020294. doi: 10.1155/2018/4020294 (PMC6151687; doi:10.1155/2018/4020294)
Supplement: Supplementary Materials — Supplementary file.docx. Table S1: information on the studied plants; Table S2: further details on the antibiotic resistance profiles of tested Gram-negative bacteria; Table S3: results of preliminary evaluation of antibiotic resistance modulatory activity of selected extracts at subinhibitory concentrations against Pseudomonas aeruginosa PA124. [file 4020294.f1.doc]

Anti-bacterial and antibiotic-potentiating activities of thirteen Cameroonian edible plants against Gram negative resistant phenotypes

Paul Nayim^a^, Armelle T. Mbaveng**^1*^**, Brice E. N. Wamba^1^, Aimé G. Fankam^1^, Joachim K. Dzotam**^1^** and Victor Kuete**^1**^**.

*^1^Department of Biochemistry, Faculty of Science, University of Dschang, Cameroon*

**Author’s addresses**

*Paul Nayim:* [*nayimpaul@yahoo.fr*](mailto:nayimpaul@yahoo.fr)

*Armelle T. Mbaveng:* [*armkuete@yahoo.fr*](mailto:armkuete@yahoo.fr)

*Elvis B. N. Wamba:* [*wambaelvis@yahoo.fr*](mailto:wambaelvis@yahoo.fr)

*Aimé G. Fankam;* [*agfankam@yahoo.fr*](mailto:agfankam@yahoo.fr)

*Joachim K. Dzotam:* [*kamgue_joachim@yahoo.fr*](mailto:kamgue_joachim@yahoo.fr)

*Victor Kuete:* [*kuetevictor@yahoo.fr*](mailto:kuetevictor@yahoo.fr)

**Corresponding authors:**

*∗ Tel.: +237 675468927; E-mail address: armbatsa@yahoo.fr (Dr A.T. Mbaveng)*

*** Tel.: +237 677355927; E-mail address: kuetevictor@yahoo.fr (Prof. Dr Victor Kuete)*

**Table S1. Informations on the studied plants**

| **Species (family); voucher number** | **Traditional uses** | **Bioactive or potentially bioactive components** | **Known antimicrobial activities of plants** |
| --- | --- | --- | --- |
| ***Azadirachta indica* A. Juss (Meliaceae); 4447/SRFK** | Diabetes [1], Respiratory disorders and constipation, control of leprosy [2], rheumatism, chronic syphilitic ,wounds and indolent ulcer [3] . | Alkaloids, glycosides, flavonoids and saponins [3] | Aqueous and ethanolic extract of barks: antimicrobial on  *Ec, Sa, Pa,* [4] , *Ec, Sa, Pa, St, Bp* [5] |
| ***Citrus grandis* (L.) (Red)**  **Osbeck (Rutaceae) ; 25860/SRFC** | The fruit peel has been used for cough, swelling, and epilepsy [6]. | The root bark contains β- sitosterol and several acridone alkaloids, and coumarins [6]. | Methonolic extracts of leaf, peel, and pulp against *Sa, Ec, St,* [6]. |
| ***Citrus grandis* (L.)(White)**  **Osbeck (Rutaceae) ; 25860/SRFC** | Leaves are reported to use in epilepsy, chorea, and convulsive cough [7]. | Alkaloids, flavonoids, saponins, terpenoids, steroids, tannins, cardioglycosides, amino acids and proteins [8]. | Ethanolic extract of leaves : antimicrobial on *Pa, Sa, Ec et Pm* [8]. |
| ***Cucurbita maxima* Duch. (Cucurbitaceae); 42449/HNC** | Urethral compliance, alleviation of diabetes, lowering the level of gastric, breast, lung and colorectal cancer [9]. | Glucids, steroids, proteins and amino acids [10]¶. ¶ | Ethanolic extract of seeds:antimicrobial on *¶Sa, Bs, Sw, Pp, Pa, Pm, Kp, Ec* [10]. |
| ***Dacryodes edulis* [G.Don] H.J.Lam (Burseraceae) ; 1874/SRFK** | Cutaneous infections and dysentery [11]. | Tanins, saponins, flavonoids, phenols, alkaloids, steroids, terpenoide, carboxylic acid, glycoside [12]. | Aqueous and ethanolic of leaves extract: antimicrobial on *Sa, Pm*, *St, Pa, Ec* [13]. |
| ***Hibiscus esculentus* L. (Malvaceae) ; 42823/HNC** | Dysentery, diarrhea, stomach and intestines irritation, Catarrhal infections of the kidneys, dysuria and gonorrhea [14]. | Flavonoids, tannins, anthracene, sterols and triterpenes [15]. | Methanolic extract and ethyl acetate extract of leaves: antimicrobial on *Bs, Sp, Kp, Sa, Ec, Pm, Pa* [16]. |
| ***Ipomoea batatas* (L.) Lam (Convolvulaceae) ; 55594/HNC** | Epilepsy, asthma, bronchitis, badly of eyes, variola, rheumatism, diarrhoea, dysentery [17] (Murugan et al., 2011); paludism, nauseas splénose, gastric distress [18].¶ | Triterpens / steroids, alkaloids, anthraquinons, coumarins, flavonoids, saponins, tanins and phenolic acid [19].. | Methanolic extract of leaves : antimicrobial on *Sa, Se, Ec, Pa, Kp, Ca, An* [20]. |
| ***Irvingia gabonensis* (Aubry. Lec. ex O. Rorke) Baill. (Irvingiaceae) ; 52936/HNC** | Hernia, yellow fever, diabetes, diarrhea, febrifuge [21]. | Tannins, saponins, alkaloids and anthraquinones and phenolic compounds [22]. | Ethanolic extracts of leaves: antimicrobial on *Sa* and *Pa* [23]. |
| ***Phaseolus vulgaris* L. (Fabaceae) ; 42587/HNC** | Sweetened diabetes ¶[24]. | Flavonoïds, tanins (derivatives of gallic acid) and saponins [25].¶ | Methanolic extract of seeds: antimicrobial on *Sa, St, Bc, Lm, Ec* [25]. |
| ***Saccharum officinarum* L. (Poaceae) ; 42958/HNC** | Arthritis, cancer, furoncles, cough, diarrhoea, dysentery, eyes, the fever, skin, laryngitis ¶ [26].  ¶ | Tannins, alkaloids, polyphenols and  flavonoïds [27]. | Hydroethanolic extract of bark: antimicrobial on *Sa, Pa and Ec* [28]. |
| ***Spondias monbin* L. (Anacardiaceae); 21249/SRFK** | ¶Diuretic, gonorrhoea, diarrhoea, dysentery, ¶hémorroïdes and leucorrhea¶ [29].¶  ¶ | Alkaloids, tanins, saponins, phenolsand flavonoids [30] ¶ | Ethanolic extract of leaves: antimicrobial on ¶*Sa, Ec, Kp, Pa* [31] (Rabbani et al., 1999).¶ |
| ***Theobroma cacao* L. (Sterculiaceae); 60111/HNC** | Diuretic, cardiotonic, detoxification, immunostimulating [32].¶ | Alkaloid, tannin, saponin, phenol, glycoside, flavonoid and carboxylic acid [32]. | Ethanolic extract of barks : antimicrobial on *Sa, Ec, Pa, Sp* [33]. |
| ***Uapaca guineensis* Muell. Arg. (Euphorbiaceae); 53136/HNC** | Fever, pain, skin diseases and sexual dysfunction [34]. | Steroids, alkaloid, terepenoids, gallic acid [35]. | Not report |

*HNC: Herbier National du Cameroun; SRFC: Société des Réserves Forestières du Cameroun; SRFK: Société des Réserves forestières du Kamerun ;* An : *Aspergillus niger, Bc* : *Bacillus cereus,* Bs *: Bacillus stearothermophilus*, Ca *: Candida albicans,* Ec *: Escherichia coli ,* Kp*: Klebsiella pneumonia,* Lm *: Listeria* *monocytogenes,* Pa*:Pseudomonas aeruginosa ,* Pm : *Proteus mirabilis,* Pp *:Pseudomonas putida.*Sa : *Staphylococcus aureus,* Se : *Staphylococcus* *epidermidis,* *Sp : Salmonella paratyphi,* St*: Salmonella typhimurium ,*Sw *: Staphylococcus werneri*.

**Table S2.** Bacterial strains and features

| **Espèces** | **Souches** | **Caractéristiques** | **Références** |
| --- | --- | --- | --- |
| ***Escherichia***  ***coli*** | ATTC 8739 | Reference strain |  |
|  | AG 100 | Wild-type *E. coli* K-12 expressing *Acr AB* efflux pumps | [36] |
|  | AG 100ATet | ΔacrAB mutant AG 100A Tet^r^ | [36] |
|  | AG 102 | AG 100 expressing *Acr AB* pumps | [37] |
|  | W 3110 | Wild-type E.*coli* K-12 | [38] |
|  | MC4100 | Wild-type E. coli K-12, KAN^R^ expressing *ABC* pump | [38] |
| ***Enterobacter aerogenes*** | ATCC 13048 | Reference strain |  |
|  | EA3 | Clinical MDR isolate CHL^R^, NOR^R^,  MOX^R^, CFT^R^, ATM^R^, FEP^R^ | [39] |
|  | EA 27 | Clinical MDR isolate exhibiting  energy-dependent norfloxacin and  chloramphenicol efflux with KAN^R^ and  AMP^R^ and NAL^R^ and STR^R^ and TET^R^ | [40, 41] |
|  | EA 289 | KAN sensitive derivative of EA27 | [39] |
|  | EA294 | EA289 expressing *AcrA* pump having KAN^r^ | [39, 42] |
|  | EA 298 | EA 289 tolC:KAN^R^ | [39, 42] |
| ***Klebsiella pneumoniae*** | ATCC11296 | Reference strain |  |
|  | K 24 | *Klebsiella pneumoniae* *AcrAB-Tolc* (Clinical laboratory collection of UMR-MD1, University of Marseille, France) | Clinical laboratory collection of UMR-MD1, University of Marseille, France |
|  | Kp 55 | Clinical MDR isolate, TET^R^, AMP^R^, ATM^R^, and CEF^R^ | [43] |
|  | Kp 63 | Clinical MDR isolate, TET^R^, CHL^R^, AMP^R^, and ATM^R^ | [43] |
| ***Providencia stuartii*** | NEA 16 | Clinical MDR isolate of *Providencia stuartii* expressing *AcrAB-TolC* | [44] |
|  | PS2636 | Clinical MDR isolate, *AcrAB* |  |
| ***Enterobacter Cloacae*** | ECCI69 | *Enterobacter cloacae* AcrAB-TolC | Clinical laboratory collection of UMR-MD1, University of Marseille, France |
| ***Pseudomonas aeruginosa*** | PA 01 | Reference strain |  |
|  | PA 124 | MDR clinical isolate *expressing MexAB-OprMpump* | [45] |

AMP^r^, ATM^r^, CEF^r^, CHL^r^, KAN^r^, NAL^r^, NOR^r^, STR^r^ et TET^r^, resistance to:ampicilline, aztreonam, céfépime, chloramphenicol, kanamycine, acidenalidixique, norfloxine, streptomycine and tétracycline respectively ;*AcrAB-TolC, MexAB-OprM :* pumps efflux.

***Table S3.*** *Preliminary evaluation of antibiotic-resistance modulatory activity of selected extracts at sub-inhibitory concentrations against Pseudomonnas aeruginosa PA124*

| **Plant extract** | **Extract concentration** | | **MIC of antibiotic (μg/mL) alone and in combination with extracts and fold increase of activity (in bracket)** | | | | | | | |
| --- | --- | --- | --- | --- | --- | --- | --- | --- | --- | --- |
|  | | | **CHL KAN STP CIP TET ERY AMP CEF** | | | | | | | |
|  | | 0 | 32 | 64 | 64 | 16 | 16 | 32 | - | - |
| **AIB** | | MIC /2 | 8 (**4**) | 16 (**4**) | 32 (**2**) | 8(**2**) | 2 (**8**) | 16 (**2**) | - (na) | - (na) |
|  |  | MIC /4 | 8 (**4**) | 32 (**2**) | 32 (**2**) | 16(1) | 2 (**8**) | 32 (1) | - (na) | - (na) |
|  |  | MIC /8 | 8 (**4**) | 32 (**2**) | 32 (**2**) | 16(1) | 4 (**4**) | 32 (1) | - (na) | - (na) |
|  |  | MIC /16 | 8 (**4**) | 32 (**2**) | 32 (**2**) | 16(1) | 4 (**4**) | 32 (1) | - (na) | - (na) |
| **CGrP** | | MIC /2 | 32 (1) | 32 (**2**) | 64 (1) | 16(1) | 8 (**2**) | 32 (1) | - (na) | - (na) |
|  |  | MIC /4 | 32 (1) | 32 (**2**) | 64 (1) | 16(1) | 8 (**2**) | 32 (1) | - (na) | - (na) |
|  |  | MIC /8 | 32 (1) | 32 (**2**) | 64 (1) | 16(1) | 8 (**2**) | 32 (1) | - (na) | - (na) |
|  |  | MIC /16 | 32 (1) | 32 (**2**) | 64 (1) | 16(1) | 16(1) | 32 (1) | - (na) | - (na) |
| **CGrL** | | MIC /2 | 32 (1) | 64(1) | 64(1) | 16(1) | 16(1) | 32 (1) | - (na) | - (na) |
|  |  | MIC /4 | 32 (1) | 64(1) | 64(1) | 16(1) | 16(1) | 32 (1) | - (na) | - (na) |
|  |  | MIC /8 | 32 (1) | 64(1) | 64(1) | 16(1) | 16(1) | 32 (1) | - (na) | - (na) |
|  |  | MIC /16 | 32 (1) | 64(1) | 64(1) | 16(1) | 16(1) | 32 (1) | - (na) | - (na) |
| **CGwL** | | MIC /2 | 16 (**2**) | 32 (**2**) | 64(1) | 16(1) | 16(1) | 32 (1) | - (na) | - (na) |
|  |  | MIC /4 | 16 (**2**) | 32 (**2**) | 64(1) | 16(1) | 16(1) | 32 (1) | - (na) | - (na) |
|  |  | MIC /8 | 32 (1) | 32 (**2**) | 64(1) | 16(1) | 16(1) | 32 (1) | - (na) | - (na) |
|  |  | MIC /16 | 32 (1) | 32 (**2**) | 64(1) | 16(1) | 16(1) | 32 (1) | - (na) | - (na) |
| **CMB** | | MIC /2 | 32 (1) | 32 (**2**) | 64(1) | 16(1) | 8 **(2**) | 64 (0.5) | - (na) | - (na) |
|  |  | MIC /4 | 32 (1) | 32 (**2**) | 64(1) | 16(1) | 8 **(2**) | 64 (0.5) | - (na) | - (na) |
|  |  | MIC /8 | 32 (1) | 32 (**2**) | 64(1) | 16(1) | 8 **(2**) | 64 (0.5) | - (na) | - (na) |
|  |  | MIC /16 | 128 (0.25) | 32 (**2**) | 64(1) | 16(1) | 16(1) | 64 (0.5) | - (na) | - (na) |
| **DEB** | | MIC /2 | 32 (1) | 32 (**2**) | 64 **(**1) | 8 **(2**) | 8 **(2**) | 32 (1) | - (na) | - (na) |
|  |  | MIC /4 | 32 (1) | 32 (**2**) | 64 **(**1) | 16(1) | 8 **(2**) | 32 (1) | - (na) | - (na) |
|  |  | MIC /8 | 32 (1) | 32 (**2**) | 64 **(**1) | 16(1) | 16(1) | 32 (1) | - (na) | - (na) |
|  |  | MIC /16 | 32 (1) | 32 (**2**) | 64 **(**1) | 16(1) | 16(1) | 32 (1) | - (na) | - (na) |
|  | |  |  |  |  |  |  |  |  |  |
| **DEL** | | MIC /2 | 64 (0.5) | 32 (**2**) | 64 (1) | 8(0.5) | 8 (2) | 32 (1) | - (na) | - (na) |
|  |  | MIC /4 | 64 (0.5) | 32 (**2**) | 64 (1) | 16(1) | 8 (2) | 32 (1) | - (na) | - (na) |
|  |  | MIC /8 | 64 (0.5) | 32 (**2**) | 64 (1) | 16(1) | 16(1) | 32 (1) | - (na) | - (na) |
|  |  | MIC /16 | 64 (0.5) | 32 (**2**) | 64 (1) | 16(1) | 16(1) | 32 (1) | - (na) | - (na) |
| **DES** | | MIC /2 | 4 (**8**) | 1 (**16**) | 1 (**32**) | 1 (**16**) | 1 (**16**) | **8 (4)** | - (na) | - (na) |
|  |  | MIC /4 | 4 (**8**) | 32 (**2**) | 32 (**2**) | 4 (**4**) | 4 (**4**) | 32 (1) | - (na) | - (na) |
|  |  | MIC /8 | 8 (**4**) | 32 (**2**) | 32 (**2**) | 4 (**4**) | 4 (**4**) | 32 (1) | - (na) | - (na) |
|  |  | MIC /16 | 8 (**4**) | 32 (**2**) | 32 (**2**) | 8 (**2**) | 8 (**2**) | 32 (1) | - (na) | - (na) |
| **HEL** | | MIC /2 | 16 (**2**) | 16 (**4**) | 16 (**4**) | 16(1) | 8 (**2**) | 32 (1) | - (na) | - (na) |
|  |  | MIC /4 | 32 (1) | 16 (**4**) | 16 (**4**) | 16(1) | 8 (**2**) | 32 (1) | - (na) | - (na) |
|  |  | MIC /8 | 32 (1) | 16 (**4**) | 32 (**2**) | 16(1) | 16(1) | 32 (1) | - (na) | - (na) |
|  |  | MIC /16 | 32 (1) | 16 (**4**) | 64 (1) | 16(1) | 16(1) | 32 (1) | - (na) | - (na) |
| **IBL** | | MIC /2 | 16 **(2)** | 16(**4**) | 64 (1) | 16(1) | 8(**2**) | 32 (1) | - (na) | - (na) |
|  |  | MIC /4 | 16 **(2)** | 32 (**2**) | 64 (1) | 16(1) | 8(**2**) | 32 (1) | - (na) | - (na) |
|  |  | MIC /8 | 16 **(2)** | 32 (**2**) | 64 (1) | 16(1) | 8(**2**) | 32 (1) | - (na) | - (na) |
|  |  | MIC /16 | 16 **(2)** | 32 (**2**) | 64 (1) | 16(1) | 8(**2**) | 32 (1) | - (na) | - (na) |
| **IGB** | | MIC /2 | 32 (1) | 64(1) | 32 (**2**) | 16(1) | 8(**2**) | 64 (0.5) | - (na) | - (na) |
|  |  | MIC /4 | 32 (1) | 64(1) | 32 (**2**) | 16(1) | 8(**2**) | 64 (0.5) | - (na) | - (na) |
|  |  | MIC /8 | 32 (1) | 64(1) | 32 (**2**) | 16(1) | 8(**2**) | 64 (0.5) | - (na) | - (na) |
|  |  | MIC /16 | 32 (1) | 64(1) | 32 (**2**) | 16(1) | 8(**2**) | 64 (0.5) | - (na) | - (na) |
| **PVL** | | MIC /2 | 16 **(2)** | 16 (**4**) | 16 (**4**) | 16(1) | 8 (**2**) | 32 (1) | - (na) | - (na) |
|  |  | MIC /4 | 16 **(2)** | 16 (**4**) | 32 (**2**) | 16(1) | 8 (**2**) | 32 (1) | - (na) | - (na) |
|  |  | MIC /8 | 16 **(2)** | 16 (**4**) | 32 (**2**) | 16(1) | 8 (**2**) | 32 (1) | - (na) | - (na) |
|  |  | MIC /16 | 16 **(2)** | 16 (**4**) | 32 (**2**) | 16(1) | 8 (**2**) | 32 (1) | - (na) | - (na) |
| **SML** | | MIC /2 | 128 (0.25) | 16 (**4**) | 64 (1) | 16(1) | 8 (**2**) | 128 (0.25) | - (na) | - (na) |
|  |  | MIC /4 | 128 (0.25) | 16 (**4**) | 64 (1) | 16(1) | 8 (**2**) | 128 (0.25) | - (na) | - (na) |
|  |  | MIC /8 | 128 (0.25) | 32 (**2**) | 64 (1) | 16(1) | 8 (**2**) | 128 (0.25) | - (na) | - (na) |
|  |  | MIC /16 | 128 (0.25) | 32 (**2**) | 64 (1) | 16(1) | 16(1) | 128 (0.25) | - (na) | - (na) |
| **SOL** | | MIC /2 | 128 (0.25) | 32 (**2**) | 64 (1) | 16(1) | 4 (**4**) | 64 (0.5) | - (na) | - (na) |
|  |  | MIC /4 | 128 (0.25) | 32 (**2**) | 64 (1) | 16(1) | 4 (**4**) | 64 (0.5) | - (na) | - (na) |
|  |  | MIC /8 | 128 (0.25) | 32 (**2**) | 64 (1) | 16(1) | 4 (**4**) | 64 (0.5) | - (na) | - (na) |
|  | | MIC /16 | 128 (0.25) | 32 (**2**) | 64 (1) | 16(1) | 4 (**4**) | 64 (0.5) | - (na) | - (na) |
|  | |  |  |  |  |  |  |  |  |  |
| **TCBB** | | MIC /2 | 16 **(2)** | 16 (**4**) | 32 (**2**) | 8(**2**) | 8 (**2**) | 16 (**2**) | - (na) | - (na) |
|  | | MIC /4 | 16 **(2)** | 16 (**4**) | 32 (**2**) | 8(**2**) | 8 (**2**) | 16 (**2**) | - (na) | - (na) |
|  | | MIC /8 | 32 (1) | 64(1) | 64(1) | 8(**2**) | 16(1) | 16 (**2**) | - (na) | - (na) |
|  | | MIC /16 | 32 (1) | 64(1) | 64(1) | 8(**2**) | 16(1) | 16 (**2**) | - (na) | - (na) |
| **TCL** | | MIC /2 | 32 (1) | 64(1) | 32 (**2**) | 8(**2**) | 8(**2**) | 8 (**4**) | - (na) | - (na) |
|  | | MIC /4 | 32 (1) | 64(1) | 32 (**2**) | 8(**2**) | 8(**2**) | 16 (**2**) | - (na) | - (na) |
|  | | MIC /8 | 32 (1) | 64(1) | 32 (**2**) | 16(1) | 16(1) | 16 (**2**) | - (na) | - (na) |
|  | | MIC /16 | 32 (1) | 64(1) | 32 (**2**) | 16(1) | 16(1) | 16 (**2**) | - (na) | - (na) |
| **UGB** | | MIC /2 | 32 (1) | 32 (**2**) | 32 (**2**) | 8(**2**) | 8(**2**) | 16 (**2**) | - (na) | - (na) |
|  | | MIC /4 | 32 (1) | 32 (**2**) | 32 (**2**) | 8(**2**) | 8(**2**) | 16 (**2**) | - (na) | - (na) |
|  | | MIC /8 | 32 (1) | 32 (**2**) | 64 (1) | 16(1) | 16(1) | 16 (**2**) | - (na) | - (na) |
|  | | MIC /16 | 32 (1) | 32 (**2**) | 64 (1) | 16(1) | 16(1) | 16 (**2**) | - (na) | - (na) |
| **UGL** | | MIC /2 | 32 (1) | 32 (**2**) | 64 **(**1) | 16(1) | 8(**2**) | 32 (1) | - (na) | - (na) |
|  | | MIC /4 | 32 (1) | 32 (**2**) | 64 **(**1) | 16(1) | 8(**2**) | 32 (1) | - (na) | - (na) |
|  | | MIC /8 | 32 (1) | 32 (**2**) | 64 **(**1) | 16(1) | 8(**2**) | 32 (1) | - (na) | - (na) |
|  | | MIC /16 | 32 (1) | 32 (**2**) | 64 **(**1) | 16(1) | 16(1) | 32 (1) | - (na) | - (na) |

^a^Samples [AIB : *Azadirachta indica* bark*,* CGrP*: Citrus grandis* (red) pericarpe, CGrL*: Citrus grandis* (red) leaves, CGwL : *Citrus grandis* (white) leaves, CMB: *Cucurbita maxima b*eans, DEB : *Dacryodes edulis* bark, DEL : *Dacryodes edulis* leaves, DES : *Dacryodes edulis* seeds, HEL*: Hibiscus esculentus* leaves, IBL : *Ipomoea batatas* leaves, IGB*: Irvingia gabonensis* beans, PVL*: Phaseolus vulgaris* leaves, SML : *Spondias monbin* leaves, SOL : *Saccharum officinarum* leaves, TCBB: *Theobroma cacao* broad beans, TCL: *Theobroma cacao* leaves, UGB : *Uapaca guineensis* bark, UGL: *Uapaca guineensis* leaves, AMP: ampicillin, CEF : cefepime; CHL: chloramphenicol, CIP: ciprofloxacin; ERY: erythromycin, KAN: kanamycin, STR: streptomycine, TET: tetracycline, −: MIC not detected at up to 256 μg/mL; (in bracket): Modulating factor; MIC Minimal Inhibitory Concentration; Values in bold represent modulating factor ≥ 2.

**References**

[1] S. K. Dholi, R. Raparla, S. k. Mankala,and K. Nagappan, “*In vivo* antidiabetic evaluation of Neem leaf extract in alloxan induced rats,” *Journal of applied Pharmaceutical science,* vol. 7, no. 4, pp. 100–105, 2011.

[2] K. R. Kirtikar, and B. D. Basu, “In: L. M. Basu. *Indian Medicinal Plants*,” 2nd edition. *Allahabad*, p. 536 pages, 1975.

[3] J. Tirumalasetty, B. Anuradha, and Praveena, “Antimicrobial activity of methanolic extracts of *Azadirachta indica, Rosmarinus officinalis* and *Lagenaria siceraria* leaves on some important pathogenic organisms,” *Journal of Chemical and Pharmaceutical Research,,* vol. 6, no. 4, pp. 766–770, 2014.

[4] P. O. Orhue, A. R. M. Momoh, E. O. Igumbor, and F. I. Esumeh, “Antibacterial effect of *Azadirachta indica* (Neem or Dongo Yaro) parts on some urinary tract bacterial isolates,” *Asian Journal of Plant Science and Research,* vol. 4, pp. 64–67, 2014.

[5] S. Maragathavalli, S. Brindha, N. S. Kaviyarasi, B. Annadurai, and S. K. Gangwar, “Antimicrobial activity in leaf extract of neem (*Azadirachta indica* Linn),” *International Journal of Science and Nature,* vol. 3, no.1, pp.110–113, 2012.

[6] A. Abirami, N. Gunasekaran, and P. Siddhuraju, “Antimicrobial activity of crude extract of citrus *hystrix* and *citrus maxima,” International Journal of Pharmaceutical Sciences And Research,* vol. 4, pp. 296–300, 2013.

[7] N. K. Dubey, R. Kumar, and P. Tripathi, “Global Promotion of Herbal Medicines: India’s Opportunity,” *Current Science*, vol. 86, no. 1, pp. 37–41, 2004.

[8] A. Jabamalairaj, S. Doraira, S. A. Yadav, and C. Bathrachalam, “Detection of functional group and antimicrobial activity of leaf extracts of *citrus grandis* (L.) against selected clinical pathogens,” *Indo American Journal of Pharmaceutical Research,* vol. 5, no. 5, pp. 2231–6876, 2015.

[9] P. Mythili and T. Kavitha, “Overview on *Cucurbita Maxima* Seed,” *Journal of Dental and Medical Sciences,*vol. 16, no. 3, pp. 29-33, 2017.

[10] K. Ravishankar, G. V. N. Kiranmayi, G. V. Appa Reddy, et al., “Preliminary phytochemical screening and *in-vitro* antibacterial activity of *cucurbita maxima* seed extract,” *International Journal of Research in Pharmacy and Chemistry*, vol. 2, no. 1, pp. 2231−2781, 2012.

[11] K. K. Ajibesin, E. E. Essien, and S. A. Adesanya, “Antibacterial constituents of the leaves of *Dacryodes* *edulis,*” *African Journal of Pharmacy and Pharmacology,* vol. 5, no. 15, pp.1782–1786, 2011.

[12] D. C. Nwokonkwo, “The Phytochemical Study and Antibacterial Activities of the seed extract of *Dacryodes edulis* (African Native Pear),” *American Journal of Scientific and Industrial Research,* vol. 5, pp. 7–12, 2014.

[13] B. A. Omogbai, and T. O. Eneh, “Antibacterial activity of *Dacryodes edulis* Seed extracts on food-borne pathogens,” *Bayero Journal of Pure and Applied Sciences,* 4(1): 17–21, 2011.

[14] T. K. Lim, “Edible medicinal and non-medicinal plants,” *Springer Science,* vol. 3, 160 pages. 2012.

[15] S. I. [Alqasoumi](https://www.ncbi.nlm.nih.gov/pubmed/?term=Alqasoumi S%5BAuthor%5D&cauthor=true&cauthor_uid=23960784), “‘Okra’ *Hibiscus esculentus* L: A study of its hepatoprotective activity,” [*Saudi Pharmaceutical J*](https://www.ncbi.nlm.nih.gov/pmc/articles/PMC3745186/)*ournal,* vol. 20, no. 2, pp. 135–141, 2012.

[16] Y. Chaudhari, E. P. Kumar, M. Badhe, H. R. Mody, and V. BAcharya, “An evaluation of antibacterial activity of  *Abelmoschus esculentus* on clinically isolated infectious disease causing bacterial pathogen from hospital,” *International journal of pharmaceutical and phytopharmacological research*, vol. 1, no. 3, pp. 107–111, 2011.

[17] D. P. Murugan, D. P. Uma, P. N. Kannika, and K. R. Mani, “Antimicrobial activity of *syzygium jambos* against selected human pathogens,” *International Journal of Pharmacy and Pharmaceutical Sciences,* 3: pp. 44–47, 2011.

[18] Milind P., Monika. “Sweet potato as a superfood,” *Pharmacology Division, Faculty of Medical Sciences, Pharmacology Division Guru Jambheshwar, University of Science and Technology*. Review Article, [www.ijrap.net](http://www.ijrap.net/), 2015.

[19] T. P. Márcia, C. F. Eliana, L. A. [Esmerino](https://www.ncbi.nlm.nih.gov/pubmed/?term=Esmerino LA%5BAuthor%5D&cauthor=true&cauthor_uid=21716926), et al., “Phytochemical screening, antioxidant, and antimicrobial activities of the crude leaves extract from *Ipomoea batatas* (L.) Lam,” [*Pharmacognosy Mag*](https://www.ncbi.nlm.nih.gov/pmc/articles/PMC3113358/)*azine,* vol. 7, no. 26, pp. 165–170, 2011.

[20] S. H. Mohamed, P. D. Hansi, and T. Kavitha, “Antimicrobial activity and phytochemical analysis of selected Indian folk medicinal plants,” *International Journal of Pharmaceutical Sciences and Research,* vol. 1, no. 10, pp. 430–434, 2010.

[21] D. J. Harris, “A Revision of the *Irvingiaceae.* *Bulletin du Jardin botanique National de Belgique*, vol. 65, no.1/2, pp. 143–196, 1996.

[22] D. A. [Fadare, and E. O.](https://www.ncbi.nlm.nih.gov/pubmed/?term=Fadare DA%5BAuthor%5D&cauthor=true&cauthor_uid=18939394)  [Ajaiyeoba,](https://www.ncbi.nlm.nih.gov/pubmed/?term=Ajaiyeoba EO%5BAuthor%5D&cauthor=true&cauthor_uid=18939394)  “Phytochemical and antimicrobial activities of the wild mango-*Irvingia gabonensis* extracts and fractions,” *African Journal of Medecine Sciences,* vol. 37, no. 2, pp.119–124, 2008.

[23] O. Nworie, J. O. Orji, U. O. Ekuma, M. V. Agah, C. S. Okoli, M. C. Nweke, “Antibacterial activity of the leaf and stem bark of *Irvingia gabonensis* (bush mango) against *Escherichia coli* and *Staphylococcus aureus*,” *Global Journal of Pharmacology,* vol. 10, no. 1, pp. 13–18, 2016.

[24] A. Helmstädter, “Beans and Diabetes: *Phaseolus vulgaris* preparations as antihyperglycemic agents,” *Journal of Medicinal Food,* vol. 13, no. 2, pp. 251–254, 2010.

[25] A. O. Teresita de Jesús, G. E. B. Aceves, M. G. R. Sotelo, and J. Y. Fernández “Methanolic extracts antioxidant and antimicrobial activities from five varieties of common beans (*Phaseolus vulgaris* L.),” *International Journal of Engineering Research and Technology,* vol. 2, no. 11, 3196–3201, 2013.

[26] J. A. Duke, “Atchley Approximate analysis. In: Christie, BR. The handbook of plant science in agriculture,” *CRC Press*, Inc. Boca Raton, FL; pp. 80–82, 1981.

[27] J. A. Mebale, F. C. Suebang, A. S. O. Azi, et al., “Chemical composition of a standard sugarcane wine of *Saccharum officinarum* Linn from Woleu-Ntem, Gabon,” *Journal of Agriculture and Sustainability,* vol. 3, no. 2, pp. 216–222, 2013.

[28] E. F. Uchenna, A. A. Okechukwu, and A. S. Chukwuemeka, “Phytochemical and antimicrobial properties of the aqueous-ethanolic extract of *Saccharum officinarum* (Sugarcane) Bark,” *Journal of Agricultural Science,* vol. 7, no. 10, pp. 291–297,2015.

[29] USDA, and ARS, “National Genetic Resources: Germplasm Resources Information Network,” *National Germplasm Resources Laboratory*, Beltsville, Maryland, 2002.

[30] A. F. Ugadu, M. C. Ominyi, M. E. Ogbanshi, U. S. Eze, “Phytochemical analysis of *Spondias mombin*,” *International Journal of Innovative Research and Development,* vol. 3, no. 9, pp. 101–107, 2014.

[31] G. H. Rabbani, M. J. Albert, H. Rahman, and A. Chowdhury, “Short chain fatty acids inhibit fluid and electrolyte loss induced by cholera toxin proximal colon of rabbit *in vivo*,” *Digestive Disease Science,* vol. 44, no. 8, pp.1547–1553, 1999.

[32] N. Singh, S. Datta, A. Dey, A. R. Chowdhury and J. Abraham, “Antimicrobial activity and cytotoxicity of *Theobroma cacao* extracts,” *Der Pharmacia Lettre*, vol. 7, no.7, pp. 287–294, 2015.

[33] D. C. Nwokonkwo, and G. N. Okeke, “The Chemical constituents and biological activities of stem bark extract of *Theobroma Cacao*,” *Global Journal of Science Frontier Research,* vol. 24, no. 4, 8 pages, 2014.

[34] B. N. Nkeh-Chungag, J. R.Temdie, C. Sewani-Rusike, Y. M. Fodjo, J. T. Mbafor, and J. E. Iputo, “Analgesic, anti-inflammatory and antiulcer properties of the extract of *Uapaca guineensis* (*Euphorbiaceae)*,” *Journal of Medicinal Plants Research*, vol. 3, pp. 635–640, 2009.

[35] E. T. Ibibia, O, Peter, and O. S. Olugbemiga, “Evaluation of some Nigerian Savannah plants of antioxydent activity, and total phenols and flavonoid contents,” *International Journal of Pharmaceutical Sciences Review and Research,* vol. 34, pp. 75-81, 2015.

[36] M. Viveiros, A. Jesus, M. Brito et al., “Inducement and reversal of tetracycline resistance in *Escherichia coli* K-12 and expression of proton gradient-dependent multidrug efflux pump genes,” Antimicrobial Agents and Chemotherapy, vol. 49, no. 8, pp. 3578–3582, 2005.

[37] C. A. Elkins and L. B. Mullis, “Substrate competition studies using whole-cell accumulation assays with the major tripartite multidrug efflux pumps of *Escherichia coli*,” *Antimicrobial Agents and Chemotherapy*, vol. 51, no. 3, pp. 923–929, 2007.

[38] P. Baglioni, L. Bini, S. Liberatori, V. Pallini, and L. Marri, “Proteome analysis of *Escherichia coli* W3110 expressing an heterologous sigma factor,” *Proteomics*, vol. 3, no. 6, pp. 1060–1065, 2003.

[39] D. Ghisalberti, M. Masi, J. M. Pages, and J. Chevalier, “Chloramphenicol and expression of multidrug efflux pump in *Enterobacter aerogenes*,” *Biochemical and Biophysical Research*

*Communications*, vol. 328, no. 4, pp. 1113–1118, 2005.

[40] M. Mallea, A. Mahamoud, J. Chevalier et al., “Alkylamino- quinolines inhibit the bacterial antibiotic efflux pump in multidrug-resistant clinical isolates,” *Biochemical Journal*, vol. 376, no. 3, pp. 801–805, 2003.

[41] M. Mallea, J. Chevalier, C. Bornet et al., “Porin alteration and active efflux: two *in vivo* drug resistance strategies used by *Enterobacter aerogenes*,” *Microbiology*, vol. 144, no. 11, pp. 3003–3009, 1998.

[42] E. Pradel and J. M. Pages, “The AcrAB-TolC efflux pump contributes to multidrug resistance in the nosocomial pathogen *Enterobacter aerogenes*,” Antimicrobial Agents and Chemotherapy, vol. 46, no. 8, pp. 2640–2643, 2002.

[43] J. Chevalier, J. M. Pages, A. Eyraud, and M. Mallea, “Membrane permeability modifications are involved in antibiotic resistance in *Klebsiella pneumoniae*,” *Biochemical and Biophysical Research Communications*, vol. 274, no. 2, pp. 496–499, 2000.

[44] Q. T. Tran, K. R. Mahendran, E. Hajjar et al., “Implication of porins in β-lactam resistance of *Providencia stuartii*,” *Journal of Biological Chemistry*, vol. 285, no. 42, pp. 32273–32281, 2010.

[45] V. Lorenzi, A. Muselli, A. F. Bernardini et al., “Geraniol restores antibiotic activities against multidrug-resistant isolates from gram-negative species,” *Antimicrobial Agents and Chemotherapy*, vol. 53, no. 5, pp. 2209–2211, 2009.
